# Addressing the causes of ‘missingness’ in healthcare: a co-designed suite of interventions

**DOI:** 10.1186/s12916-026-05031-3

**Published:** 2026-06-26

**Authors:** Calum Lindsay, David Baruffati, Mhairi Mackenzie, David A. Ellis, Michelle Major, Catherine A O’Donnell, Sharon A Simpson, Geoff Wong, Andrea E Williamson

**Affiliations:** 1 General Practice and Primary Care, School of Health and Wellbeing, University of Glasgow, Glasgow, UK; 2 Department of Urban Studies, School of Social & Political Sciences, University of Glasgow, Glasgow, UK; 3 Centre for Healthcare Innovation and Improvement Information, Decisions and Operations, Centre for Business Organisations and Society (CBOS), University of Bath, Bath, UK; 4 Homeless Network Scotland, Glasgow, UK; 5 Public Health, School of Health and Wellbeing, University of Glasgow, Glasgow, UK; 6 Nuffield Department of Primary Care Health Sciences, University of Oxford, Oxford, UK

**Keywords:** Missed appointments, Non-attendance, Missingness, Access to care, Health inequalities, Access inequalities

## Abstract

**Background:**

Missingness refers to ‘a repeated tendency not to take up offers of care that has a negative impact on the person and their life chances’, visible in patterns of missed health appointments. Epidemiological work has shown that patients experiencing ‘missingness’ are more likely to have multiple physical and mental health conditions, to live in adverse or precarious circumstances, and experience a range of negative health outcomes. Yet existing approaches designed to address missed appointments rarely focus on these patients; when they do, it is often through a punitive lens rather than one that engages meaningfully with the causes of missingness. As a result, existing interventions are often ineffective for these patients and may instead worsen access inequalities. This study addresses this gap by outlining a co-produced ‘suite’ of interventions aimed at addressing the specific, complex causes of missingness.

**Methods:**

The study synthesised findings from three co-occurring workstreams: an extensive realist review of 253 documents including peer-reviewed and grey literature; interviews with 61 ‘key informants’ whose personal and professional experiences provided insight into causes and possible solutions; and a series of co-design workshops with a Stakeholder Advisory Group of 16 professionals and experts-by-experience aimed at designing and refining an intervention.

**Results:**

The intervention consists of activities across several key domains: embedding a change of perspective around missed appointments; identification; relationships and communication; missingness coordinators; transport and logistics; flexibility; and contact around appointments. Unifying these activities, and crucial to their success, is a paradigm shift towards missed appointments that we term a ‘missingness lens.’

**Conclusions:**

This intervention presented here is an evidence-informed, realistic and meaningful set of actions with the potential to address the complex causes of missingness and, by extension, access inequalities and wider health inequalities. Future interventional research into missed appointments should actively focus on or include ‘missing’ patients in design, implementation and the measurement of outcomes.

**Supplementary Information:**

The online version contains supplementary material available at 10.1186/s12916-026-05031-3.

## Background

Missed appointments have become central to policy discourse around healthcare globally [1]. In multiple country contexts, health services generally—and primary care in particular—are impacted by overlapping crises of growing demand and limited capacity [2]. Demand comes through aging populations with increasingly complex health needs, societal mental health crises, and a rise in poverty alongside the erosion of social safety nets [3, 4]. Capacity is diminished by chronic underfunding; shortages of appropriate staffing and infrastructure; unsustainable workloads and staff burnout; and delays, and thus services that appear unresponsive and inaccessible [2, 5, 6]. In this context, policymakers often frame missed appointments as a growing source of wasted money and lost capacity, and addressing them becomes a financial, moral, and even existential imperative [7, 8]. Policy, research and practice typically seek cost-effective reductions in missed appointments *for services* by ‘fixing’ problematic patient behaviours, rather than changing how services are designed and delivered [9].Where patients miss *multiple* appointments, the individual behavioural lens produces stigmatising and Othering narratives of patients as “repeat offenders” [10, 11], “chaotic”, or “irresponsible” [12, 13]. The circumstances of ‘missing’ patients’ lives, evidenced in preceding epidemiological work [14–17], also attracts stigmatising narratives around poverty, substance use, mental health and long-term conditions that are also often ascribed to individual failings [18–20].

Yet as our prior work has shown, ‘missingness’—“the repeated tendency not to take up offers of care that has a negative impact on the person and their life chances” [21]—has a range of complex, overlapping and mutually reinforcing causes that occur at several points on patients’ journeys to and through health services [15]. These include: the sense that healthcare is not ‘for me’—not necessary, beneficial, appropriate, or safe; past experiences of stigma, discrimination, neglect and abuses of power, as well as trauma and relational adversity; exposure to competing demands and crises, with fewer resources to address them; issues managing official ‘gatekeeping’ systems or the unwritten rules of engagement; travel, transport, and difficulties moving safely to and through healthcare spaces; and an overall sense of mistrust and distrust [15]. Societal and institutional structures play a significant role. Stigma influences whether people possess the resources needed to negotiate care and patterns encounters with healthcare and other social institutions [22p.1]. Healthcare is unequally distributed and is often a poor ‘fit’ with people’s health experiences and life circumstances [23].

These experiences are rarely reflected in the design of interventions because they are absent from existing evidence about them. Focused on benefits to services, research is rarely stratified by who is impacted and how—and who may be missing [24, 25]. People are excluded by recruitment criteria—for example, those with poor mental health, cognitive impairment, learning disabilities, with insufficient English language skills, without phones, with incomplete practice records, or who are otherwise deemed unsuitable [26–29]. Few studies reflect on self-selection bias and those patients less likely to accept or use the intervention [30]. These issues are structurally patterned, with those excluded often at greater risk of access issues or negative outcomes. Lacking a phone may suggest particular adversity [31p.53]; missing administrative data may reflect access issues, exclusion, or mis/distrust [24, 32 p.7]. The limited evidence that does consider patients likely to be ‘missing’ shows that they are either least likely to benefit from or are actively disadvantaged by interventions designed to ‘work’ for all patients [24, 33, 34]. Some studies consider cancellation as a positive outcome as it reduces the overall unexpected non-attendance rate [35–37] but this still represents a missed opportunity for care and few studies explore *who* cancels, nor whether patients are simply deterred from seeking care in the first place [24]. Thus ‘missing’ patients also experience “structural missingness” [38], their knowledge and experiences absent from conversations about access to healthcare. This paper aims to address this by reporting on a study that synthesised extensive primary and secondary data to design a suite of co-produced, evidence-informed interventions to address missingness in primary care.

## Methods

The intervention development approach was guided by realist principles and by the Six Steps in Quality Intervention Development (6SQuID) approach [39]. Here we focus on the first four steps: defining and understanding the problem; identifying malleable factors which have scope for change; identifying how to bring about change (the active ingredient or change mechanism); and exploring how to deliver the change mechanisms. The final two stages involve implementing and evaluating the intervention and are not covered here due to funding parameters but will be the focus of future work. Interventions designed through 6SQuID involve “changing relationships, displacing existing activities and redistributing and transforming resources” [39]. They use a systems lens to recentre the role of systemic and contextual causal factors, which are often absent from predominantly behavioural approaches [40].

The stages of the 6SQuID process were addressed through the iterative synthesis of data from the three overlapping workstreams of the “developing interventions to reduce ‘missingness’ in health care” project. The study design was approved by the University of Glasgow College of Medical, Veterinary and Life Sciences Ethics Committee in 2022 (Reference 00220187). Workstream 1 was a realist review of literature, identified through database searching and citation-tracking (see [41] for full methods) complemented by further citation tracking and ‘umbrella’ reviews of intervention ideas identified in other workstreams. A full PRISMA diagram and list of included documents are available in Additional File 1 (Figure S1, Table 1). Workstream 2 consisted of realist interviews with 61 key informants whose personal and professional experiences brought a broad range of clinical, social and inclusion health perspectives on missingness. Given our interest in including voices or perspectives absent from existing research, we approached partner organisations whose staff, volunteers and service users might fill these gaps. We took practical steps to overcome barriers to participation: travelling to participants throughout the UK; giving the choice of remote interviews; arranging translated documents and interpreters; compensating participants; and working in a manner that built trust and security between participants and researchers [42].

Participant recruitment began on 28th February 2023 and concluded on 11th July 2024. All participants provided informed consent in writing or, where this was not possible, consent was provided verbally and audio-recorded prior to interview. Interviews were ‘realist’ in that they focused on participants’ theories about the causes of missingness, actions that might address these causes, and factors contributing to or inhibiting their success (see [23] and [22] for full methods). By asking participants to reflect on our emerging programme theory, and to bring their own experiences and perspectives to bear on the drivers of missingness and interventions to address it, interviews addressed many of the gaps and shortcomings in the research literature. A breakdown of interview participants is available in Additional File 1 (Table S2).

Workstream 3 consisted of a series of Stakeholder Advisory Group (StAG) workshops. The StAG had 16 members (8 experts-by-experience and 8 professionals) who reflected a broad range of patient and professional perspectives in the key areas of inclusion health; mental health; homelessness; asylum and migration; substance use; and “severe and multiple disadvantage” [43]. The group met regularly throughout the project, initially acting as guides and sense-checkers for the other workstreams, then as co-designers of an overall interventional approach [44]. All members of the StAG were also interviewed in workstream 2. Participants in the StAG process provided informed consent in writing to participate in this process.

The contribution of each method to the 6SQuID approach is outlined in Table 1, and the materials used to guide stakeholder discussions are included in Additional File 1 (Sect.  6).

**Table 1:** Contribution of workstreams to the 6SQuID process

**1. Defining and understanding the problem.**
**Review**: Despite limitations, there is a body of critical literature providing an alternative to the dominant ‘problematisation’ of missingness [45] and a set of ‘evidential fragments’ [46] to build on. These provided empirical, conceptual and theoretical foundations for our theory of missingness.
**Interviews**: Helped address the gaps in the existing evidence base, brought insights from groups seldom heard in prior research, and provided nuanced understanding of the complexity of missingness including a contextual and structural understanding of the problem absent from the literature.
**StAG**: Provided guidance and a ‘sense-check’ for our understanding of the problem, helping refine, amend and address gaps. Also guided for recruitment strategies for interviews, and our approach to analysis and synthesis of the existing research evidence.
**2. Identifying malleable factors which have scope for change.**
**Review**: Outlined a refined programme theory of the key causes of missingness and those factors considered amenable to change, organised into key domains [15].
**Interviews**: Provided further depth and breadth of insight into the refined programme theory, particularly relating to wider social structures and contextual conditions [23].
**StAG**: Validated the refined programme theory, then engaged in discussion of initial intervention ideas to address different causes at different ‘levels’ of the social-ecological model [47].
**3. Identify how to bring about change (active ingredient/change mechanism).**
**Review and Interviews**: Provided a set of intervention for discussion at the StAG. A further umbrella review looked in detail at several intervention domains identified by StAG members but which were not explored fully in the initial review, and additional literature added further detail to other domains.
**StAG**: Explored intervention activities and grouped them into domains linked to the causes they address. Using hypothetical patient case studies, the group considered which interventions might address the constellation of causes evident for each patient.
**4. Exploring how to deliver the change mechanisms**.
**Review**: Analysis of research literature helped provide details of contextual conditions for intervention success in each domain, along with barriers, preconditions and key considerations for successful implementation.
**Interviews**: Provided crucial contextual information from real-world examples of interventions: promising activities, key elements for successful implementation, and possible blockers or barriers.
**StAG**: The group outlined a set of core principles crucial to the success of intervention activities across different contexts (a ‘missingness lens’). Through practice-level case studies, they explored resource requirements, challenges, drawbacks, risks, and unintended outcomes.

Moving back and forth between workstreams allowed each to address the limitations of the others. For example, interventional literature is characterised by significant heterogeneity of settings or approaches to the same activity, *and* by a lack of contextual description, making assessment of implementation processes barriers, risks or unintended outcomes difficult [48–50p.5]. These were then explored in interviews and stakeholder workshops. Similarly, the alternative version of the problem evident in interviews and StAGs helped identify additional causes at interpersonal, institutional, community and structural levels, and thus alternative intervention activities for exploration in the iterative literature review process.

The process of analysis was similarly recursive. Literature was analysed in NVivo, with data relevant to the causal working and contextual dynamics of interventions extracted and synthesised into an explanatory narrative in potential intervention domains. Interviews were analysed separately in NVivo, coded abductively according to a theoretical framework combining fundamental causation theory and the candidacy framework (see [23] and [22] for further details). Findings from the literature were refined further by findings from interviews, and both used to create materials presented to and refined further by StAG members. Audio recordings and visual materials from StAG meetings were summarised and synthesised with discussions from previous meetings into a final intervention. The final suite of interventions detailed here was synthesised by the research team using summary documents from all three workstreams and was validated by participants at the final StAG. Given the quantity of the data collected, and the nature of the synthesis process above, results are presented as a narrative without primary or secondary data specifically referenced. The contribution of each work package to each intervention domain is described instead in Additional file 1 (Table S5). Primary data are available in the final project report [51] and are available on the Open Science Framework [51], congruent with the principles of open research.

## Results

### Key principles: a missingness lens

**Fig. 1:**
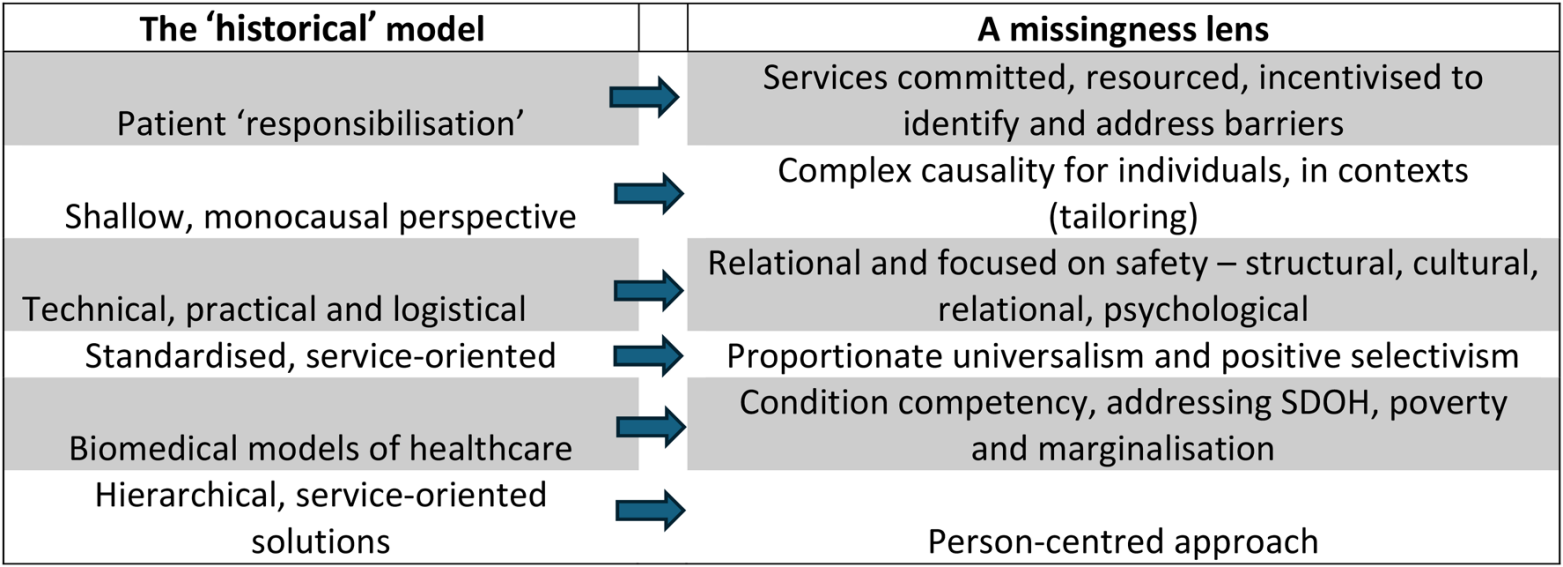
The historical model and the ‘missingness’ lens

The intervention presented here is more accurately a ‘suite’ of interventions, or a “recyclable core set of processes” that can be applied according to the requirements of different contexts [52]. Their successful implementation depends on creating “congruence” [52] between each part by rooting them within a set of principles termed ‘the missingness lens’ (see Fig. 1). These principles were defined during a StAG meeting, and they represent a fundamental shift in how the ‘problem’ of missingness is understood and how solutions to it should be designed and implemented.

Firstly, instead of considering missingness as a problem for services caused by patient behaviour, a missingness lens means services commit to directing resources to addressing missingness as a problem for patients and their health. Rather than designing, implementing and measuring interventions across an unstratified patient population, a missingness lens involves both “proportionate universalism” [53]—shifting resources towards those in greatest need—and “positive selectivism” [54] by targeting specific patients and evaluating outcomes for them. It means moving from shallow, simple explanations towards deep and complex causes at multiple levels: structural influences on patients’ circumstances and resources; institutional dynamics of service provision; and interpersonal and intrapersonal processes. It also means moving beyond logistical, technical or practical solutions by recentring affective, psychological and relational elements, particularly around *safety -* psychological safety through trauma-informed practice and psychologically-informed environments [55]; and cultural and structural safety by identifying and addressing sources of stigma, discrimination and power imbalance [56]. This includes a central role for patient-centred, collaborative care. Finally, a missingness lens moves beyond biomedical and clinical approaches to understand health and healthcare in their wider context through condition competency—“clinical understanding of the lived-experience of specific conditions” [23]—and structural competency, the ability to recognise and to influence the social and structural determinants of health [57].

### A ‘suite’ of interventions

**Fig. 2:**
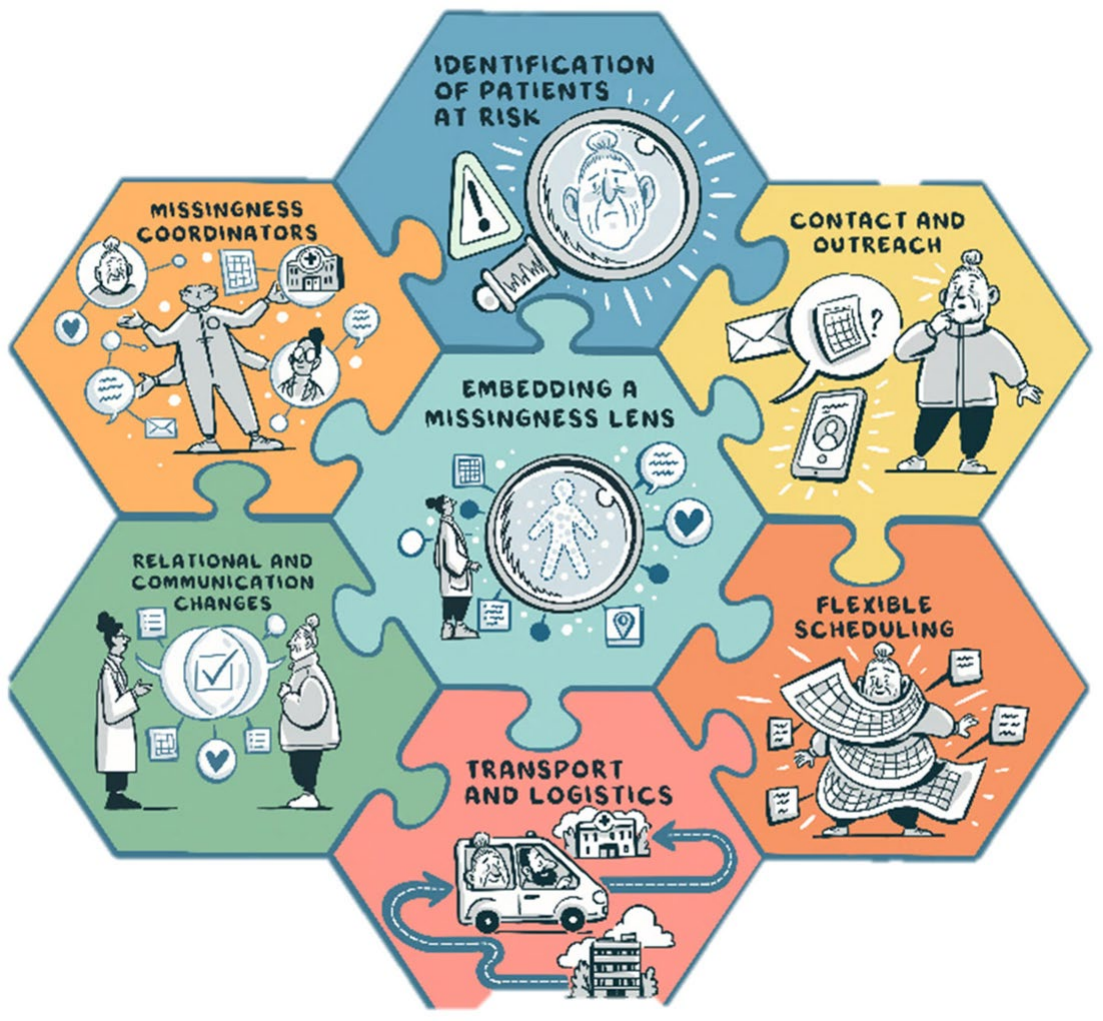
The co-designed ‘suite’ of interventions

The specific suite of actions outlined below represent an attempt to disrupt the systemic causal dynamics sustaining missingness, either directly or by pursuing intermediate outcomes in support of this goal [40, 58]. They are limited to those actions which health services have the power to change, while cognisant of the need for broader policy changes to address social determinants of missingness and of illness. The actions are split into several interconnected domains but are likely to be most impactful when synthesised rather than as a set of discrete elements. The domains are represented in Fig. 2 below, followed by detailed discussion of how each can be implemented in a way that is congruent with the missingness lens.

### Embedding a missingness lens

The first step in addressing missingness is in embedding the elements of this perspective shift at all levels of health service organisation and delivery. Participants often described the need for a “culture change” towards missed appointments at multiple levels—clinical, administrative, policy, planning, and funding. Resources including money, time, staffing, data infrastructure, knowledge and skills need to be mobilised and prioritised to ensure this culture change is matched with concrete changes in practices and systems. At the broadest level, this means services having the money to ensure that missingness-focused work—likely to be complex, and time- and labour-intensive—is supported, encouraged and even incentivised. Many participants felt that current NHS funding structures like the Quality and Outcomes Framework (QOF) actively disincentivise missingness-related work, with “exception-reporting” used to exclude patients from monitoring and reporting, rather than incentivising work to get patients into practices. Without specific funding, even the best intentions would remain a “fantasy” or a “dream.” Funding should permit localised innovation and plans for making and monitoring change through multi-component quality improvement methods: setting clear and transparent goals, planning and carrying out key actions, and regularly reviewing or amending them through inclusive, proactive systems for accountability. Bringing together staff and patient perspectives collaboratively is a crucial part of this process. Targeted action, built on solid principles and localised planning, matched with resources (staffing, knowledge, financial) acts as the foundation upon which the activities below can be built. Monitoring and accountability mean going beyond service-side metrics of missed appointment rates to measure impacts on patients qualitatively and quantitatively. They should also occur above individual care delivery, at the strategic and policy level.

Several other actions are suggested to support the embedding process. The first involves training for staff, using knowledge and skills-development to “displace” [39] existing perspectives—particularly stigmatising attitudes or opinions among staff towards people experiencing missingness and particular health-related or help-seeking behaviours. Training should be provided to all staff at all levels – from administrative to clinical to commissioning—to ensure congruence and consistency. In theory, raising awareness of the negative health outcomes around missingness, of its main causes, and of actionable solutions, may lead to increased staff motivation and empathy, to buy-in for meaningful action, and, for patients, the sense that services are supportive and safe. All three workstreams suggested other, complementary areas for professional development, including poverty and inclusion health, stigma and discrimination, mental health, psychological trauma, neurodivergence, and adult attachment. Training should be designed to help staff reflect on practice systems and on relationships and communication dynamics: how and why patients might present differently; how to manage challenging encounters; and how to enact principles of relational care and to create safe environments. Expertise-by-experience is important in formulating and delivering training activities to drive awareness, empathy and change.

However, focusing exclusively on staff attitudes to embed the missingness lens risks simply reversing the direction of blame without acknowledging staff who are motivated to change practice but are inhibited by limited resources and capacity and the burnout they cause. Knowledge, skills and motivation need to be matched by concrete resources to turn motivation into meaningful action. Staff need the protected time and capacity to engage with training and development, and providers the capacity to allow staff to attend as well as the access to training resources and expertise. To allow the missingness lens to displace existing practices, training needs to be complemented by supervision and reflective space, positive reinforcement and feedback, and ongoing support for staff. There is also a role for utilising existing staff knowledge and experience of missingness. Staffing changes may be appropriate, including actively recruiting new clinical and non-clinical staff from communities identified as being at risk of missingness, who may bring knowledge of barriers to care and may act as a bridge to those communities.

### Identification of patients at risk

To build a localised plan for missingness, and to monitor its success, practices and services require IT infrastructure and data recording systems to monitor missingness trends and outcomes. Services need systems to identify who is missing through retrospective analysis of appointment trends, and to identify and flag those who become missing in real-time. Past work has demonstrated some difficulties in using UK primary care systems in this way [14, 17, 59]. Practices should prioritise the creation of robust coding of patient records, particularly of non-attendance, and should seek to address missing data which is often a site of inequality [38]. Services can complement this with other ways to identify those who are missing, for example notifications of missingness from referrals to secondary or tertiary care, or staff knowledge of the patient population. While recently ‘machine learning’ approaches have been designed to play this role, caution is needed due to concerns about inaccuracy, bias and the absence of a critical approach to causes in these systems [9].

In carrying out identification, services must decide on their thresholds for missingness, particularly considering current resource environments. The initial epidemiological work on missingness selected those with two or more missed appointments over a three-year period, based on a pilot study and stakeholder group validation [14, 17, 60]. Missingness impacted 19% of the total patient population – a cohort of patients with complex health and social needs [14, 16, 17]. Uniformly applying this intervention to all these patients simultaneously risks diluting available resources and limiting impact. To resolve this, services may elect to focus only on those who miss the greatest number of appointments, or whose patient profiles suggested particular vulnerabilities. Crucially, this work is dynamic and recursive and can change as projects develop, and as ideas are tested and approaches refined over time.

Services should complement pattern-identification with identification of the causes of missingness, carried out by contacting those experiencing it and having non-judgemental, respectful and invitational conversations about their experiences. These conversations may have instrumental benefits in identifying individual and collective causal patterns, and may also represent the first step in a change in relationships [39]. Staff become more aware and understanding of patients’ circumstances; patients feel a sense of care and effort on the part of services that may previously have seemed uncaring or exclusionary. More broadly, connections with key community stakeholders, community groups or settings may also improve identification of access barriers among specific groups. Within a Quality Improvement (QI approach, regular monitoring and feedback inputs from staff, individual patients, and across the practice population on areas relevant to missingness acts as a point of ongoing identification.

The findings from identification can be used in two ways. Firstly, they can feed into broader practice changes aimed at improving access to care. Secondly, they can underpin an approach to positive selectivism and proportionate universalism, where particular patients or groups are targeted for tailored responses and additional resources [53, 61]. This is closer to individual *needs* identification, covering the specific and proximate causes of missingness in a collaborative and person-centred way, actively seeking and building on patients’ priorities and perspectives, and resulting in a concrete and tailored set of actions from services. A Patient Individual Needs (PIN) document could be created, detailing a patient’s key information and priorities; their access needs and communication preferences; and the steps to be taken by services to support them. Services may also seek insight from significant people or services in patients’ lives (with their consent). The findings of these assessments become the foundations of consistency, continuity and accountability, reducing patients’ needs to advocate for access or additional support, or to repeatedly disclose sensitive information to staff in their pursuit of care. Stakeholders felt that this document should be NHS-wide, ensuring consistency across the system. In line with a person-centred approach these assessments and the support following from them should not be limited to clinical or access issues. Possessing few resources to manage competing demands in multiple domains is a central cause of missingness; if not identified and addressed, these causes of missingness and of ill health are likely to endure [41].

### Relational and communication changes

All workstreams explored the importance of relationships in addressing missingness, and the centrality of trust, consistency, continuity and safety for patients whose service interactions have often been patterned by stigma, discrimination, exclusion, neglect, even hostility. A missingness lens involves recentring the relational causes of missed appointments and including relational components throughout the intervention – for example, the role of training in relational care and trauma-informed, non-stigmatising practice in embedding a missingness lens, or the relational work of reaching out to patients to identify their needs. The outcome of these actions, and the core of a relational approach, is creating consistency and continuity at every stage of the patient journey, from registration to consultation and follow-up. Patients’ anxieties or fears will likely be reduced if they can trust in a response that is consistently welcoming, invitational, non-judgemental and supportive, not reprimanding, punitive, coercive or manipulative. For those with profoundly negative relational histories, this is even more important. This is not to say that these relationships are unboundaried—instead, clear, agreed boundaries and reciprocal expectations are central to consistency.

Several concrete actions are suggested here. Extending efforts at continuity of care to ‘missing’ patients is central. Being able to see a familiar clinician may reduce anxieties about attendance and increase feelings of safety and security and may also create a sense of reciprocity or loyalty and a relationship to be protected by attending. Being familiar to a clinician or key staff member may encourage “condition competency” [23] and “structural competency” [62] and care plans built upon patients’ needs, circumstances and preferences. Continuity does not have to mean seeing the same person at every interaction. A dedicated small team of clinical and non-clinical within practices focused on missing patients may provide sufficient continuity and retain some flexibility for practices. If patients can contact practices with the knowledge of who they might speak to and how they might be received, by staff aware of their circumstances and needs, anxieties around gatekeeping and initial access may be reduced. Other concrete actions include identifying and catering to patients’ communication needs and preferences in all interactions (e.g. language barriers, literacy issues, cognitive challenges, communication preferences).

Relational work takes time. Given the levels of mistrust and past trauma often evident in missingness, people may not instantly respond to relationship-building work. Identification-work will often require a relationship to be established over time, so that the individual feels safe and able to disclose important information about themselves. Persistence is central, but there is also a need to respect patients’ right to not be involved, and to avoid being overly persistent, coercive or manipulative, or perpetuating problematic power dynamics. A patient-centred and collaborative approach to the intervention and to clinical practice, coupled with a non-judgemental, supportive approach, may contribute to a sense that care is ‘for me’ both instrumentally and relationally.

### Missingness coordinators

The additional work required to address missingness brings the question of *who* is best positioned to do it. Addressing missingness effectively requires a ‘missingness coordinator’, a role akin to other non-clinical roles (e.g. navigators, care coordinators, peer workers, mentors, community outreach workers, ‘Focused Care’ workers [72]) but oriented specifically around missingness. Missingness coordination involves several key tasks. Firstly, coordinators carry out identification-work and build relationships with patients, seeking to develop trust through active, empathetic, non-judgemental and collaborative support. Coordination is a core part of relational continuity and consistency, a reliable point of contact for the patient and an advocate for them and for good relational practice. In this, missingness coordinators engage in bridging, brokering or mediating activities aimed at creating “safe passage” [63] to and through services. Bridging activities may include working with patients to build their personal resources—confidence, motivation, self-esteem, even expectations for health—as well as supporting changes in service provision through individual, institutional and systemic advocacy, such as the advocacy against austerity carried out by Deep End GPs [64]. Care needs to be taken to avoid these workers being made solely responsible for missingness, or their work being marginalised or even co-opted by existing problematic practices. This requires that coordinators be skilled and well-supported, and that they be embedded in services in ways that allow them to change practice in line with the missingness lens.

Using a holistic, person-centred approach to identification, coordination extends beyond primary care settings. It may involve coordinating healthcare across multiple service settings to reduce treatment burden, and coordinating with other key services (e.g. housing, benefits, justice, social care, domestic abuse) to address social determinants of illness and the precarity that impedes good health and healthcare access. The shape of the work will vary from patient to patient, and thus coordination requires flexibility, open-endedness and a tailored and person-centred approach.

Additional considerations include who is best placed to act as a coordinator, and how they might be funded or positioned to maximise their impact. Expertise-by-experience and ‘peer-ness’ may provide a route to credibility, relationship-building and cultural and structural safety, although stakeholders and interviewees felt that coordinators can come from a range of personal and professional backgrounds as long as they are sufficiently trained, skilled and supported to engage with complex and difficult circumstances. This, plus the need to ‘embed’ workers as equal partners, suggested that paid staff were more appropriate than volunteers. While NHS funding may provide a route to embeddedness, third-sector independence may be a virtue for those mistrustful of the healthcare system.

### Flexible scheduling

Given that inflexibility or ‘impermeability’ of services are a central cause of missingness [15], addressing it means changing practice and service systems to be more flexible and suitable for different patient circumstances. There is strong support across all strands of work for prioritising ‘missing’ patients for different forms of tailored flexibility. This includes flexibility in how appointments are made – in person, online, outside the usual appointment-making windows, bypassing reception gatekeeping or telephone triage, booking via coordinators or directly with ‘missingness’-focused staff who can facilitate access. Offering greater choice in when appointments occur may maximise convenience and minimise interference from other demands, as may offering appointments outside of regular hours to those experiencing barriers at other times. Prioritising missing patients for rapid access, drop-in access or open appointment slots may allow providers to minimise delays or take advantage of the “window of opportunity” [24] that exists at the point where care is sought. Offering longer appointments, underpinned by person-centred consultation styles, may have multiple benefits – more time and space to build rapport, for patients to describe needs, circumstances, concerns or preferences, and to discuss and negotiate proposed courses of action. These also become an opportunity to maximise the impact of each single appointment, and thus the perceived benefits of attending in future. Similarly coordinated care planning, or having the flexibility to combine appointments with other health or non-health service providers, may increase the value of attendance and minimise the burdens of managing multiple engagements.

Alongside temporal flexibility, services should offer flexibility in *who* patients see (see relationships and communication above) and in *where* patients are seen. This may mean a role for home visits, or for outreach/in-reach into key settings where identification work suggests ‘missing’ patients are likely to be found (e.g. homelessness services, alcohol and drug recovery services, community organisations). In the early stages of missingness work, patients may feel safer and more secure in a familiar environment and their transport and logistical issues may be resolved. Outreach or in-reach may build the initial relational links that lead to more regular engagement or contribute to word-of-mouth and the engagement of other ‘missing’ patients. Remote care through telephone or video appointments is also an option: patients may feel safer or more secure in their home environments, and travel costs, time or difficulties may be eliminated, as may competing demands like work or childcare.

Flexibility also means making allowances or accommodations for lateness and avoiding punitive or exclusionary responses to non-standard presentation or continued non-attendance. The shape of flexibility and its limits can be discussed and agreed with patients during initial identification, to ensure both they and service providers have a set of clear expectations and boundaries, agreed and recorded in the PIN. Crucially, it should be offered actively and gladly, neither guarded nor gatekept, as this may demonstrate a level of care and commitment on the part of the service that patients respond to - but only if carried out through a missingness lens without which interventions risk being ineffective or exclusionary. Consider telephone or video appointments, where many of the causes of missingness endure and new causes emerge: a lack of access to technology, limited tech literacy, changing contact details, the costs of data or internet access, complex or unreliable systems, mistrust and concerns about privacy or data security, the lack of safe space, the loss of relational immediacy and limitations on rapport and communication. Carried out with an embedded missingness lens, and designing systems with and for ‘missing’ patients, mean these systems are likely to ‘fit’ patients’ preferences or circumstances. This may include using easily accessible systems, ensuring communication preferences are addressed, and providing support through coordinators to access or use devices or data, connect to systems and facilitate communication. Finally, there is a role for coordinators facilitating flexibility beyond primary care, where ‘missing’ patients might be prioritised to overcome access barriers in secondary and tertiary care including long delays, requirements for re-referral after missed appointments, and exclusionary eligibility criteria.

### Transport and logistics

As well as being an aspect of flexibility, changing the site or the mode of an appointment is a logistical intervention aimed at reducing barriers related to travel – costs and time, reliance on support networks or unreliable transport systems, limited mobilities, poor mental health, feelings of unsafety [15]. There is a spectrum of possible actions here, their suitability dependent on the circumstances of patients and the resources available to practices. At the least intensive end, practices may consider reimbursing travel costs, although this depends on patients having sufficient money to cover costs initially. Services may pre-emptively provide tickets, tokens, vouchers or parking permits instead. Facilitated access to NHS or voluntary transport services may also be beneficial. At the more intensive end, taxi transport and home visits may be most appropriate, as may accompaniment by a missingness coordinator to support safe “wayfinding” [65]. Accompaniment can also mean supporting patients within consultations—supporting the exchange of information, providing reassurance or advocacy, and reducing anxieties or worries about attending. Systems for arranging transport need to be accessible, inclusive, and actively offered, as often they are described as inaccessible, complex, inflexible and either actively gatekept or implicitly patterned by eligibility criteria or narratives of deservingness that deter access. Changing service spaces using trauma-informed principles, or setting aside quiet or private waiting rooms, are also crucial to ensuring people feel safe and secure.

### Contact and outreach

Rather than a narrow domain of ‘reminders’, our evidence suggests an expansive approach to patient contact around appointments. Reminding has a role, but reminders alone will not address deeper, broader and more enduring barriers than the simple forgetfulness that most systems aim to address. Basic SMS, automated or email reminders are a minimum standard—the medium determined by patients’ contact preferences, with messages including details of the appointment (time, location, staff member, even purpose). “Stepped” reminders [24] may be beneficial—multiple messages, perhaps sent using different methods, with one reminder far enough in advance to allow patients to plan and other closer to the time to ensure patients do remember. Patients’ preferences should again guide this action. Concerns about data security, privacy and confidentiality should be discussed and addressed where possible—and where patients express a preference to avoid reminders, this should be respected. Practices should prioritise systems for patients to respond to reminders with minimal barriers in order to discuss, cancel or rearrange their appointments. They should work to obtain patients’ contact details and preferences on practice systems, and to maintain these systems to account for changes, otherwise these systems risk perpetuating inequalities. Patients should have the option to identify significant others to contact if they are unavailable.

Yet simply increasing the intensity or quantity of reminders, or tweaking wordings or formats, are unlikely to address deeper causes. There is no support here for behavioural ‘nudges’ in contact around appointments, which were seen to be stigmatising, coercive and alienating. Instead, services should explore personalised pre-appointment contact with identified patients. Personalised contact, carried out by coordinators, may help the relationship-building process with an invitational, welcoming and supportive approach. It may help support orientation as staff and patients discuss what to expect, explore concerns and potential benefits, and identify barriers that are then addressed by actions in other intervention domains.

Contact around appointments also includes contact after appointments, particularly if they are missed, as an opportunity to express care and concern, maintain connection and to check in on the patient’s wellbeing and any health needs or unaddressed access barriers. This should be framed as an invitational offer of support, rather than as a reprimand. The approach to contact should be outlined during identification and agreed in the PIN, so patients know they will be contacted and who will be contacting them, by what method, and with what purpose. Crucially, contact around appointments needs to be two-way. Patients must have the means to respond to written reminders to query or amend details or discuss any concerns. There also needs to be room for patient-initiated contact between or around appointments, whether via clinical teams, dedicated contact lines, or through coordinators, otherwise contact systems risk perpetuating asymmetries where services can freely contact patients but the patient cannot contact them in return.

### Other approaches

Several possible actions are not included here. Some are excluded because stakeholders did not discuss them or because they saw limited value or active drawbacks to using them. Incentivisation of attendance (financial, in kind, or contingency management) did not feature strongly, perhaps reflecting concerns about making healthcare transactional rather than relational, and perpetuating power dynamics and trust. There was no support for fines, punishments or sanctions, or for overbooking approaches that are essentially punitive and were described by one StAG member as “sinister.” Other approaches are promising but fall beyond the purview of individual services – such as ‘hub’ models and integrated services, bringing multiple support services into spaces and thus increasing coordination and reducing the burdens of accessing multiple services. Other aspects are simply beyond the power of health services to change – poverty, austerity, poor housing, the migration system, stigmatising policy discourse, and other determinants of illness – although these continue to act as major impediments to care and to good health. The health service does retain an influencing and advocacy role here, however, as the site where many of the ill effects of broader policy are often most visible.

## Discussion and conclusions

This paper outlines the main components of a major paradigm and practice shift about multiple missed appointments in primary care, based on the application of a missingness lens and of key resources targeted at addressing the complex causes of missingness. The suite of interventions outlined here represent a core set of activities to be applied flexibly according to local contextual factors. A comprehensive approach to missingness would include all the proposed actions, but we recognise the constraints of current primary care systems and the need to prioritise some patients, and some actions, until resourcing permits a more expansive approach. Prioritising the cultural shift of the ‘missingness lens’ is crucial, as are the twin processes of identification and of relational and communication change through which practices might better understand their patients’ challenges and the ways in which their own service delivery processes may contribute to inequity. In turn, this can support services to build more equitable systems of care in the other domains.

This study has several strengths. Faced with a literature base built without key perspectives, causal insights or theories of change, our stakeholders and interviewees brought alternative (and seldom-heard) understandings and theories from their direct, contextualised experiences [66]. Using knowledge from within systems to build an intervention increases its relevance, practicality and feasibility, and thus its potential for success [39, 67]. While there was a high degree of congruence between workstreams, areas of disagreement between data sources could often be accounted for by the flaws of the literature base, or simply by difference of experience, context, preference or positionality between participants. What ‘works’ for one person may not work for another. These differences illuminate, rather than obscure, important causal and contextual dynamics, and highlight the importance of a tailored approach. A further strength relates to the relevance of this work beyond primary care, as both the primary and secondary data include evidence from across the healthcare system.

Although this study is evidence-informed and based on thorough theorising, it does not test or evaluate the proposed intervention in practice. However, it does provide some implications for future research and evaluation of interventions into missingness and missed appointments more generally. Some we have detailed previously—the need to change the framing of missed appointments, to include data and analysis on missingness, and to use inclusive methods of recruitment and data collection [15]. Ensuring data is equally available for ‘missing’ patients, acknowledging where and why it is not, and stratifying analysis of intervention effects according to how they impact those experiencing missingness, are crucial ways to counter “structural missingness” [38]. Interventional research should also carefully consider what outcomes are measured, with a stronger focus on patient health and wellbeing and on patients’ *chosen* outcomes in a person-centred approach. It should not only measure numerical reductions in missed appointments, or in missingness, but should also explore intermediate outcomes related to causes: do patients feel cared for, supported and understood? Is their experience of care or its quality improved? Does their health or quality of life improve? Crucially, how are these improvements unequally distributed? Finally, greater reporting of the context and process of interventions is crucial to improving an evidence base where these insights are limited.

A further limitation of this study relates to its ‘level’ of focus. During intervention design, contributors considered interactions within and between multiple levels of focus – structural, community, institutional, individual—before focusing on changes within the power of health services. Consequently, many causal factors fall beyond our remit, particularly structural factors: growing poverty and inequality, austerity politics, crumbling transport infrastructure, the decimation of the third sector safety net; hostile immigration policies, and the systematic underfunding of health and social services that erode their capacity and erode public trust within them. If these issues continue to worsen there is a risk that institutional and practice changes become “fantasy paradigms” unable to mitigate deeper and more powerful forces [68].

The suite of interventions outlined here are highly congruent with several recent papers on addressing access and health inequalities in the UK [61, 69–71]. Beyond specific Inclusion Health settings such as homelessness health care there are already encouraging mainstream care examples, such as the Inclusion Health Action in General Practice (IHAGP) approach in Scotland. ‘Deep End’ practices were resourced to address inequalities, with missingness chosen as a point for action. Many practices embedded a missingness lens and changed their communication practices through staff development in trauma-informed care, cultural competence and communication; carried out identification through proactive outreach to patients; and offered flexibility through extended consultations [59]. While these changes delivered improved experiences for ‘missing’ patients and for staff, concerns remain about sustainability without continued funding and support [59]. The unique contribution of the work to date is that missingness provides a tangible and measurable focal point for work to address both access inequalities and broader health inequalities. These problems are often framed as being particularly complex and intractable, overwhelming and demoralising to those seeking to address them. Addressing missingness provides an anchor point, a framework and a site for meaningful change, and with the application of resources to the problem evidence suggests that profound transformation can be achieved [59, 63, 72–74]. The next stage for this ‘missingness’ research is to systematically test the suite of interventions presented here, and to fully explore what difference the missingness lens can make in the current context.

## Supplementary Information

Below is the link to the electronic supplementary material.

Supplementary Material 1: Additional file 1: Figure S1, Tables S1-S5

## Data Availability

The literature review datasets generated and/or analysed during the current study are available in supplementary files and the Open Science Framework ‘Developing interventions to reduce ‘missingness’ in health care 10.17605/OSF.IO/E4BDV. 55 interview transcripts can be accessed on request via an email to the corresponding author or to shw-missingness@glasgow.ac.uk. The following conditions apply: new users will not attempt to de-identify research participants, new users will emulate the values of the original research purpose and not seek to stigmatise individuals or communities, and no UK or international laws will be broken. Due to the sensitive disclosive nature of interviews, some of the transcripts are closed to access. One interview was not transcribed.

## Abbreviations

NHS: National Health Service
GP: General Practice/General Practitioner
6SQuID: 6 Steps in Quality intervention Development
PRISMA: Preferred Reporting Items for Systematic Reviews and Meta-Analyses
StAG: Stakeholder Advisory Group
QI: Quality Improvement
PIN: Patient Individual Needs
IHAGP: Inclusion Health Action in General Practice
